# Clustering Based on Innate Immunity Reveals Differential Dysregulation Based on Disease Severity in Myelodysplastic Neoplasms

**DOI:** 10.1002/hon.70104

**Published:** 2025-05-21

**Authors:** Pedro Robson Costa Passos, Andréa Alcântara Vieira, Renata Pinheiro Martins de Melo, Ronald Feitosa Pinheiro Filho, Leonardo Guimarães Sampaio, Hermano Vinnicius Gomes dos Santos, Letícia Rodrigues Sampaio, João Victor Caetano Goes, Sílvia Maria Meira Magalhães, Ronald Feitosa Pinheiro

**Affiliations:** ^1^ Laboratory of Cancer Cytogenetics Federal University of Ceará Fortaleza Brazil; ^2^ Research Center for Drug Development (NPDM) Fortaleza Brazil; ^3^ Postgraduate Program in Medical Sciences Federal University of Ceará Fortaleza Brazil; ^4^ Postgraduate Program in Pathology Federal University of Ceará Fortaleza Brazil; ^5^ Department of Clinical Medicine Federal University of Ceará Fortaleza Brazil

**Keywords:** gene expression, innate immunity, irak kinases, myelodysplastic syndromes, toll‐like receptor

## Abstract

Myelodysplastic neoplasms (MDS) are clonal hematologic disorders characterized by ineffective hematopoiesis and a variable risk of progression to acute myeloid leukemia (AML). Despite growing recognition of the role of innate immunity in MDS pathogenesis, the precise mechanisms remain unclear. In this study, we analyzed bone marrow CD34+ expression data from 183 MDS patients to investigate the impact of the Toll‐like receptor (TLR) pathway on disease progression. Six key innate immunity genes (*IRAK1*, *IRAK2*, *IRAK4*, *MYD88*, *TRAF6*, and *NFKB1*) were used to define two distinct immune clusters: a hyperactive immune cluster (HIC) and a moderate immune cluster (MIC). The HIC was enriched in 155 immune‐related pathways and showed higher infiltration of activated natural killer cells and M1 macrophages, while the MIC exhibited increased infiltration of naïve B cells and mast cells. Differential expression analysis identified 35 genes that distinguished the clusters. Validation in an independent cohort of 82 patients revealed that reduced expression of these genes correlated with markers of advanced disease, including lower hemoglobin levels, lower neutrophil counts, altered cytogenetics, and higher bone marrow blast percentages. These findings underscore the critical role of immune dysregulation in MDS progression and highlight novel therapeutic opportunities within the innate immunity pathway for tailored interventions.

**Abbreviations**
AML: Acute Myeloid Leukemia
AML‐RM: Acute Myeloid Leukemia Related to Myelodysplasia
BMPCs: Bone Marrow Pooled Cells
Cq: Quantification Cycle
DEGs: Differentially Expressed Genes
GSEA: Gene Set Enrichment Analysis
HIC: Hyperactive Immune Cluster
HSPC: Hematopoietic Stem and Progenitor Cells
IL: Interleukin
IRAK: Interleukin‐1 Receptor‐Associated Kinase
KEGG: Kyoto Encyclopedia of Genes and Genomes
MDS: Myelodysplastic Neoplasms
MIC: Moderate Immune Cluster
NF‐κB: Nuclear Factor kappa B
TLR: Toll‐Like Receptor

## 1 Introduction

Myelodysplastic neoplasms (MDS) constitute a heterogeneous group of clonal hematologic disorders characterized by ineffective hematopoiesis and an increased risk of acute myeloid leukemia (AML). The pathogenesis of MDS is linked to recurrent somatic mutations, resulting in the displacement of normal hematopoietic stem and progenitor cells (HSPCs) by their clonal counterparts [1, 2, 3]. Although the clonal dominance of MDS HSPCs has already been well elucidated, the mechanisms driving this phenomenon remain uncertain [4, 5]. Mutations associated with clonal hematopoiesis and MDS are increasingly recognized in earlier stages of life, raising questions about their sufficiency in disease development [6, 7]. This has shifted some of the focus toward understanding the selection mechanisms rather than the initial development of mutated HSPCs [2]. The inflammatory profile of the bone marrow microenvironment has emerged as a selective pressure influencing the survival and expansion of these clonal populations [2, 3, 4, 5].

MDS HSPCs exhibit a unique innate immune response, with more than 50% of patients showing mutations and gene overexpression related to innate immune pathways [5, 8]. This leads to heightened inflammatory signaling characterized by increased levels of cytokines, chemokines, microbial signals, and alarmins [5]. While inflammation typically impairs HSPC self‐renewal in chronic infections [9], in MDS, it plays a dual role by both contributing to bone marrow failure through HSPC cycling and promoting a proliferative phenotype that benefits clonal expansion [2, 3, 4, 5, 10]. MDS HSPCs are also known to overexpress toll‐like receptors (TLRs), particularly TLR2 and TLR4, and generate mutated macrophages that have increased NOD‐like receptor protein 3 inflammasome activation and interleukin (IL) 1 beta production, triggering the noncanonical nuclear factor kappa B (NF‐κB) pathway [2, 3, 11]. We previously demonstrated a positive correlation between interleukin 8 (IL‐8) and NF‐κB levels in MDS patients, suggesting that NF‐κB may drive IL‐8 expression and contribute to the inflammatory environment [12].

Despite promising results from preclinical studies targeting innate immunity in MDS [13], gaps remain in understanding how the TLR and NF‐κB pathways are related to MDS pathogenesis. While TLRs have been extensively studied, comprehensive investigations focusing on downstream signaling factors crucial for elucidating how they affect prognosis are lacking [11, 14]. It is also unclear if any of the underexplored immunity‐related genes may serve as effective targets for inducing pro‐apoptotic phenotypes [3, 15]. To address these gaps, we analyzed how clinical and molecular data from MDS patients were affected by innate immunity dysregulation.

## 2 Subjects and Methods

### 2.1 Ethical Approval

The study received approval from the local ethics committee (process Nº 76021523.2.0000.5054).

### 2.2 Data Sources

We first analyzed data from the GSE19429 dataset available in the NCBI Gene Expression Omnibus (GEO) database, which includes mRNA expression data from the bone marrow CD34+ cells of 183 MDS patients. We subsequently examined samples collected from two reference Brazilian hospitals. This in‐house cohort aimed to address the limitations of the GSE19429 dataset, which lacked key disease‐specific parameters. Our cohort included 82 individuals: 76 diagnosed with MDS and 6 with acute myeloid leukemia myelodysplasia‐related (AML‐MR). For this group, we examined the mRNA expression data of bone marrow pooled cells (BNPCs) samples, along with peripheral blood, cytogenetic parameters, and bone marrow biopsy. For the characteristics of the in‐house cohort, see Supporting Information S1: Table S1. Given the extensive research on TLRs, we directed our attention to six pivotal downstream signaling genes: *NFKB1*, *TRAF6*, *MYD88*, *IRAK1*, *IRAK2*, and *IRAK4*.

### 2.3 Clustering Analysis

Using data from GSE19429, we utilized the “ConsensusClusterPlus” package from R software to assess the ideal number of clusters and then performed k‐means clustering on log2‐transformed gene expression data of our six assessed genes (*NFKB1*, *TRAF6*, *MYD88*, *IRAK1*, *IRAK2*, and *IRAK4*).

### 2.4 Differentially Expressed Genes and Gene Set Enrichment Analysis

Differentially expressed genes (DEGs) were screened between the clusters (FDR < 0.05, |log_2_FC| ≥ 1). Protein‐protein interactions (PPI) between their products were assessed using the string database (https://www.string‐db.org/). We conducted gene‐set enrichment analysis (GSEA) between the clusters to identify which pathways are differentially functional between the groups. A Reactome pathway analysis was used to explore the TLR pathway, complemented by Kyoto Encyclopedia of Genes and Genomes (KEGG) enrichment analyses to investigate additional pathways of interest [16, 17]. The R package “clusterProfiler” was used to screen for KEGG pathways (NES| > 1, Nom *p*‐value  <  0.05).

### 2.5 Immune Infiltration Estimation

The CIBERSORT gene signature (http://cibersort.stanford.edu/) was used to infer immune cell types in the selected clusters. This feature employs a computational approach that accurately resolves relative fractions of diverse cell subsets in gene expression profiles from complex tissue. The scores of infiltrating immune cells were calculated using this method, enabling an in‐depth analysis of the tumor microenvironment [18].

### 2.6 Cytogenetic Analysis

Bone marrow was collected in heparin under sterile conditions and divided into two vials containing 7 mL of RPMI 1640 medium (pH 7.0), 3 mL of fetal bovine serum, and 100 μL of L‐glutamine. The material was cultured for 24 h in an incubator at 37°C with 5% CO_2_. Banding was performed via the trypsin technique, and the bands were stained with Giemsa [19]. At least 20 metaphases from each patient were analyzed via a computerized system with CytoVision software. The results were reported according to the International System for Human Cytogenomic Nomenclature (ISCN) criteria.

### 2.7 Real Time RT‐PCR

Total cellular RNA was extracted from BNPCs of patients with MDS and AML‐RM via TRIzol (Invitrogen, Carlsbad, CA) according to the manufacturer's protocol. cDNA synthesis was performed via the High Capacity cDNA Reverse Transcription Kit (Applied Biosystems, Carlsbad, CA) following the manufacturer's recommendations. The quantification of gene expression was performed via real‐time PCR on a 7500 Real‐Time PCR System (Applied Biosystems, Carlsbad, CA). Three reference genes (*ACTB*, *GAPDH*, *HPRT1*) were identified via the online tool RefFinder (https://www.heartcure.com.au/reffinder/). Results were evaluated via Sequence Detection System v1.3 software (Applied Biosystems, Carlsbad, CA) to obtain quantitative cycle (Cq) values. We then calculated the ΔCq values and 2^−ΔCq^ for both the target and reference genes.

### 2.8 Statistical Analysis

For comparing gene expression values within different groups, we employed either Nonparametric Mann Whitney test or Kruskal‐Walles test with Dunn's post hoc tests, assuming the Bonferroni correction for multiple comparisons. For categorical variables, we employed the Chi‐squared test. For risk stratification in the in‐house cohort, patients with a revised International Prognostic Scoring System (IPSS‐R) classification of very low, low, or intermediate risk were categorized as having early‐stage disease, while those with high, very high risk, or AML‐MR were classified as having advanced‐stage disease. Statistical analyses were performed in R (version 4.3.2, R Foundation for Statistical Computing, Vienna, Austria), with two‐sided tests and a significance threshold of *p* < 0.05.

## 3 Results

### 3.1 Clustering and Immune Infiltration

**FIGURE 1:**
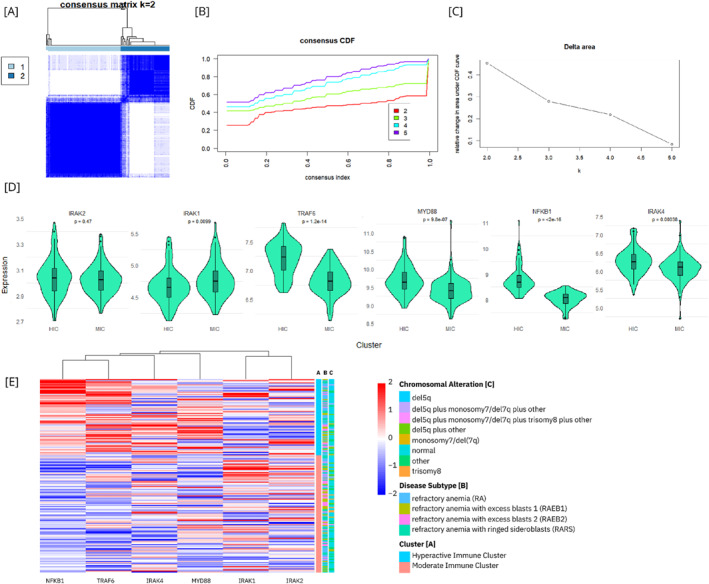
Clustering‐based division of MDS patients based on innate immunity. [A] Consensus clustering analysis identified two distinct patient clusters. [B] Cumulative distribution function illustrating cluster stability across two to five clusters. [C] Relative changes in the area under the cumulative distribution function curve as the number of clusters increases. [D] Differential expression of six key genes across the identified clusters. [E] Integration of gene expression differences with chromosomal alterations, disease subtypes, and cluster assignments. CDF, consensus distribution function; HIC, hyperactive immune cluster; MIC, moderate immune cluster.

**FIGURE 2:**
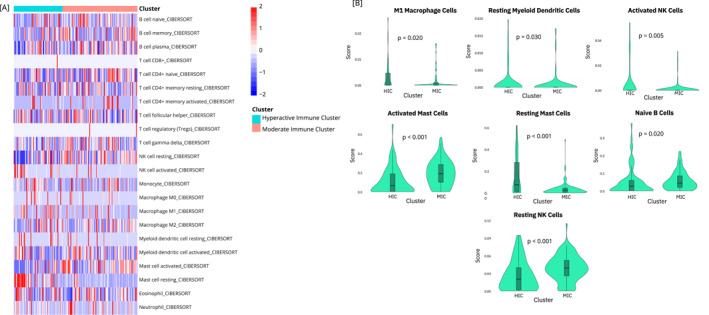
Immune assessment by CIBERSORT deconvolution within MDS patients. [A] Mapping of the distributions of immune cell‐related scores stratified by cluster assignment. [B] Significant differences of immune cell expression between clusters. HIC, hyperactive immune cluster; MIC, moderate immune cluster; NK, natural killer.

Consensus clustering of the expression patterns of six key genes (*NFKB*, *TRAF6*, *MYD88*, *IRAK1*, *IRAK2*, and *IRAK4*) identified an optimal number of clusters at *k* = 2 (Figure 1A‐C). 72 patients (39.3%) were grouped into a cluster characterized by elevated expression of *NFKB1*, *TRAF6*, *MYD88*, and *IRAK4*, designated as the “Hyperactive Immune Cluster” (HIC, Figure 1D) due to its overexpression of four out of the six assessed genes. The remaining 111 patients (60.7%) were assigned to a cluster with increased expression of *IRAK1*, referred to as the “Moderate Immune Cluster” (MIC, Figure 1D). No significant differences in chromosomal alterations or disease subtypes were observed between these clusters (*p* = 0.326 and 0.346, respectively; Figure 1E). Immune infiltration estimation analysis revealed that the HIC was enriched for M1 macrophages, resting myeloid dendritic cells, activated natural killer (NK) cells, and resting mast cells (*p* = 0.020, 0.030, 0.005, and < 0.001, respectively), whereas the MIC showed higher levels of naive B cells, active mast cells, and resting NK cells (*p* = 0.020, < 0.001, and < 0.001, respectively, Figure 2A–B).

### 3.2 Pathway Assessment

**FIGURE 3:**
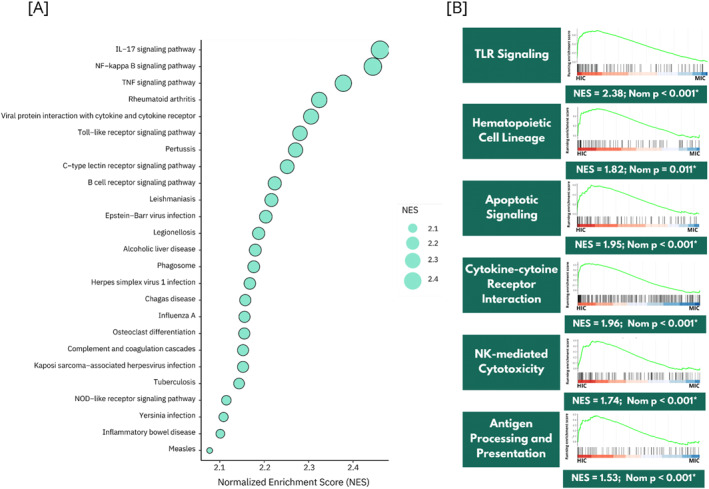
Gene set enrichment analysis of the hyperactive immune cluster in comparison to the moderate immune cluster. [A] Top 25 enriched pathways in the hyperactive immune cluster, ranked by normalized enrichment score. [B] Normalized enrichment score and nominal *p*‐value of key immune‐related pathways relevant to MDS. NES, normalized enrichment score; Nom *p,* nominal *p*‐value.

GSEA identified 155 enriched pathways in the HIC compared to the MIC. Figure 3A displays the top 25 enriched pathways. The HIC demonstrated significant enrichment in pathways relevant to MDS and innate immunity: the TLR pathway (normalized enrichment score [NES] = 2.42, *p* < 0.001, Figure 3B), hematopoietic cell lineage (NES = 1.80, *p* < 0.001, Figure 3B), the apoptotic signaling pathway (NES = 1.97, *p* < 0.001, Figure 3B), cytokine‐cytokine receptor interaction (NES = 1.93, *p* < 0.001, Figure 3B), NK cell‐mediated cytotoxicity (NES = 1.71, *p* < 0.001, Figure 3B), and antigen processing and presentation (NES = 1.55, *p* = 0.012, Figure 3B).

### 3.3 Differentially‐Expressed Genes

**FIGURE 4:**
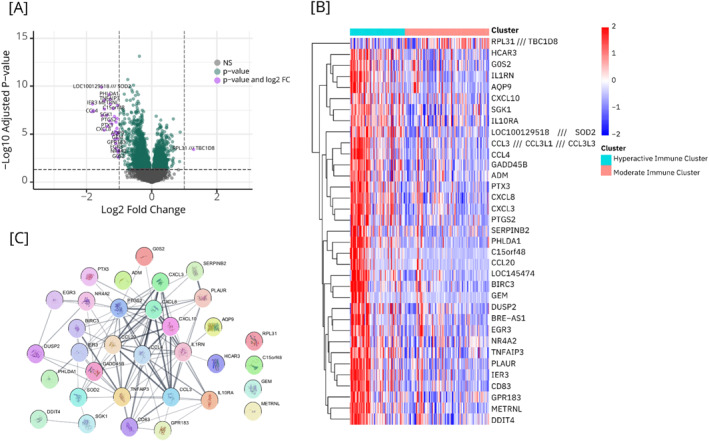
Differentially expressed gene analysis between the clusters. [A] Volcano plot highlighting differentially expressed genes filtered by *p*‐value < 0.05 and Log_2_(Fold Change) > 1. [B] Cluster‐specific mapping of the identified differentially expressed genes. [C] Protein‐protein interaction network showing known interactions among proteins encoded by the differentially expressed genes. FC, fold change, NS, non‐significant.

We identified 35 DEGs between the two clusters, with only one gene, *RPL31*, showing higher expression in the MIC cluster (Figure 4A–B). For specific adjusted *p*‐values and log fold‐change, see Supporting Information S1: Table S2. PPI analysis revealed significant interactions among the DEGs (Figure 4C).

### 3.4 Peripheral Blood Parameters

**FIGURE 5:**
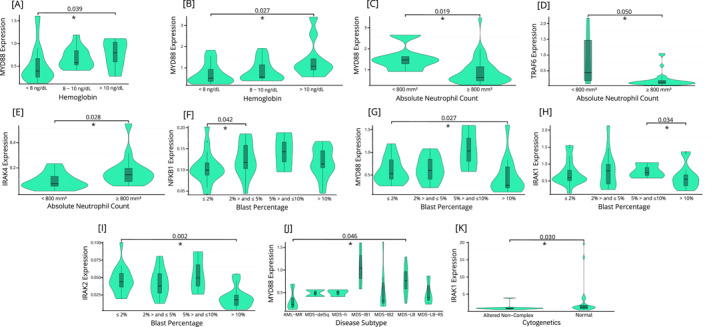
Significant associations among surrogate outcomes of advanced disease by expression of six key genes in the in‐house cohort. [A], [F], [G], [H], [I], and [J] represent analyses conducted across all patients. [B], [C], [D], and [K] focus on early disease patients, while [E] examines advanced disease patients. Comparisons involving more than three factors were analyzed using the Kruskal‐Wallis test with Dunn's post‐hoc adjustment. Comparisons between two factors were performed using the Wilcoxon test. AML‐MR, acute myeloid leukemia myelodysplasia‐related; MDS‐del5a, myelodysplastic neoplasm with deletion of 5q; MDS‐h, hypoplastic myelodysplastic neoplasm; MDS‐IB1, myelodysplastic neoplasm with increased blasts 1; MDS‐IB2, myelodysplastic neoplasm with increased blasts 2; MDS‐LB, myelodysplastic neoplasms with low blasts; MDS‐LB‐RS, myelodysplastic syndromes with low blasts and ring sideroblasts.

Patients from the in‐house cohort were split into groups for gene expression analysis on the basis of the IPSS‐R thresholds for hemoglobin (< 8 g/dL, 8–10 g/dL, and > 10 g/dL), absolute neutrophil count (ANC; < 800 mm^3^ and ≥ 800 mm^3^), and platelet count (< 50,000; 50,000 ‐ < 100,000; and ≥ 100,000). Patients with hemoglobin levels below 8 g/dL had lower *MYD88* expression than did those with levels above 10 g/dL (*p* = 0.039; Figure 5A). Early disease (see methods for definition) patients maintained this trend (*p* = 0.027, Figure 5B). Individuals within early disease with ANC of < 800 mm^3^ had significantly higher expression of both *MYD88* and *TRAF6* when compared to those with ≥ 800 mm^3^ (*p* = 0.019 and *p* = 0.050, respectively, Figure 5C–D). Advanced disease patients with ANC of < 800 mm^3^ overexpressed *IRAK4* when compared to patients with ≥ 800 mm^3^ (*p* = 0.029, Figure 5E).

### 3.5 Bone Marrow Parameters

Patients from the in‐house cohort were grouped by blast percentage using IPSS‐R thresholds: ≤ 2%, > 2% – < 5%, 5%–10%, and > 10%. *NFKB1* expression was significantly lower in patients with ≤ 2% blasts than those with > 2 – < 5% (*p* = 0.042, Figure 5F). *MYD88* expression was significantly lower in patients with > 10% blasts than in those with ≤ 2%, blasts (*p* = 0.027; Figure 5G). *IRAK1* expression was lower in patients with > 10% blasts than in those with 5%–10% blasts (*p* = 0.034; Figure 5H). For *IRAK2*, patients with > 10% blasts had reduced expression relative to those with ≤ 2% blasts (*p* = 0.002; Figure 5I). No significant associations were observed between gene expression and dyserythropoiesis, dysmegakaryopoiesis, or dysgranulopoiesis (See Supporting Information S1: Table S3).

### 3.6 Disease Subtype

We selected disease subtypes with more than one patient in our cohort for this analysis. All were classified on the basis of the 5th edition of the World Health Organization Classification of Haematolymphoid Tumors [20]. Our analysis revealed that *MYD88* expression was significantly greater in AML‐RM patients than in MDS‐LB patients (*p* = 0.046, Figure 5J).

### 3.7 Cytogenetic Parameters

Patients from the in‐house cohort were grouped both by cytogenetic results (normal, altered non‐complex, and complex) and using IPSS‐R classifications, with those with very good, good or intermediate prognostic value being classified as low cytogenetic risk, and those with bad or very bad risk being classified as high cytogenetic risk. The only significant association was seen in early disease patients, in which those with normal cytogenetics had higher *IRAK1* expression when compared to those with altered non‐complex cytogenetics (*p* = 0.030, Figure 5K).

## 4 Discussion

Altered expression across various levels of innate immune signaling drives distinct biological effects in MDS [11, 21, 22]. Low‐risk MDS is characterized by a hyperimmune state, with increased cytotoxic T lymphocyte and helper T cell 17 targeting MHC‐class I molecules on HSPCs, longside a significant decrease in regulatory T cells that facilitates the apoptosis of mutated HSPCs [15, 23]. This activation is often driven by the NF‐κB pathway through overexpression of activators like TLR2 and TLR4 and downregulation of inhibitors such as miR145 and miR146a [3, 23, 24, 25]. In contrast, high‐risk MDS is typically associated with an anti‐apoptotic phenotype, with upregulation of the anti‐apoptotic protein Bcl‐2 and a reduction in apoptotic cell markers like Apo2.7 in CD34+ cells [15, 26]. In our analysis, the HIC exhibited an enrichment of apoptosis‐related pathways, along with a heightened NK cell activity, an increase in M1 macrophages, differential upregulation of immune‐related genes, and enrichment of various immune pathways compared to the MIC, consistent with features associated with low‐risk MDS [3, 21, 23, 27, 28, 29]. In this sense, HIC's immune activation may enhance early disease surveillance but also contribute to bone marrow failure. Conversely, the MIC phenotype may reflect late immune exhaustion or suppression, potentially facilitating disease progression and resistance to certain immunotherapies.

Stratifying the MDS spectrum is helpful for assessing target populations for novel treatment. Preclinical studies have explored all of our assessed gene products with the exception of IRAK2 [14, 30, 31, 32]. However, indirect NF‐κB targeting was the first to advance into human trials for MDS, with the proteasome inhibitor Bortezomib showing modest and variable response rates [33]. Inhibition of NF‐κB enhances apoptosis in MDS blast cells and is thought to play a critical role in the transition from high‐apoptotic to low‐apoptotic phenotypes [27]. The therapeutic effects of Myd88 and Traf6 inhibition are believed to stem from the counteracting of the anti‐apoptotic functions of NF‐κB [14, 34]. Notably, the only trial targeting NF‐κB in MDS patients has focused on low‐risk patients [33], which may account for their only moderate outcomes, as the induction of apoptosis may have greater effects on anti‐apoptotic phenotypes [30]. IRAK4 inhibitors have also entered human trials, with Emavusertib, a selective IRAK4 inhibitor, currently being evaluated in the Phase 1/2 TakeAim Leukemia trial (NCT04278768), which has shown promising preliminary efficacy [35].

High *MYD88* expression has been paradoxically associated with both reduced overall survival and low‐risk disease in MDS patients [14]. Interestingly, inhibiting MYD88 in CD34+ cells of low‐risk MDS patients has been shown to increase erythroid colony formation [14]. Given the known dynamics between hyperimmuninity and hematopoiesis, *MYD88*‐enriched MDS link to HSPC dysfunction is likely due to chronic TLR signaling [24, 36, 37]. However, we found that higher hemoglobin levels were associated with increased *MYD88* expression, a trend that persisted when stratifying by early, but not advanced, disease. This pattern may indicate that *MYD88*‐based signaling predominates in the early stages of the disease, before HSPC dysfunction strongly impacts hemoglobin levels. Indeed, AML‐MR patients exhibited lower *MYD88* expression than MDS‐LB patients. *MYD88* is a potent stimulator of IL‐8, IL‐6, and IL‐1 signaling [14, 38, 39, 40], which are implicated in chronic erythroid dysfunction [12, 41, 42, 43, 44]. Furthermore, because our analysis included BMPCs rather than isolated CD34+ cells, the observed effects might reflect *MYD88'*s broader influence on the hematopoietic niche. *MYD88* signaling influences macrophage differentiation, CD4+ T‐cell production of IFN‐γ, and both early and late hematopoiesis [45, 46, 47, 48, 49].

Early disease patients with lower ANCs also exhibited *MYD88* overexpression. We hypothesize that reduced ANCs may drive *MYD88* overexpression rather than the reverse, as *MYD88* plays a critical role in facilitating TH1 differentiation in response to immune challenges [46, 50], which these patients are more prone to. This could explain the increased mortality in *MYD88*‐enriched patients [14]. Of note, higher *TRAF6* expression was also linked to decreased ANCs in early disease patients. Although this may follow the mechanisms previously described, as *TRAF6* is an important downstream mediator of *MYD88* signaling, previous studies have shown that the loss of *TIFAB*, a gene associated with MDS‐del5q, results in increased TRAF6 levels and altered myeloid differentiation, including neutrophil dysplasia and cytopenia [3, 51]. Furthermore, *TRAF6* overexpression is linked to the ubiquitination of hnRNPA1, which affects RNA processing and contributes to hematopoietic defects [52].

Research on IRAK expression in MDS remains limited, with most attention focused on *IRAK4*, whereas other IRAK family members have not been extensively evaluated [53, 54]. We observed that *IRAK1* and *IRAK2* underexpression were associated with higher bone marrow blast percentages. This same effect was seen in *MYD88*, meaning that much of the pathway is underexpressed in this group, with the exception of *NFKB1*, in which overexpression correlated with high blast percentage. This exception is likely due to the dependency of blast survival and proliferation on an anti‐apoptotic environment [55, 56]. *IRAK1'*s association in early disease with normal cytogenetics, a surrogate marker for overall survival and leukemic transformation in MDS [57, 58], also reflects the protective role of hyperimmunity in preventing malignant transformation. Moreover, advanced disease patients with higher ANCs exhibited overexpression of *IRAK4*. Upon activation, *IRAK4* stimulates the production of pro‐inflammatory cytokines and chemokines [59, 60, 61]. Due to this, *IRAK4*‐inactive mouse models exhibit reduced inflammatory responses, including diminished neutrophil recruitment [62]. Therefore, IRAK4 inhibitors may adversely affect ANCs, particularly in high‐risk patients whose immune responses are already compromised [15]. Given that IRAK4 inhibitors have not yet been evaluated in high‐risk MDS, this raises a significant concern for their potential impact in this population.

This study has limitations that should be acknowledged. First, the in‐house cohort was derived from a single country, which may limit the generalizability of our results. Second, the use of BMPCs could obscure cell‐specific expression patterns, although this has shown to not significantly impact pathway analysis [63]. Third, the study did not include functional validation of the identified patterns, leaving the mechanistic implications to be inferred from correlative data. Fourth, the absence of a well‐defined low‐immune cluster reduces interpretability. Finally, we were unable to incorporate potentially relevant patient data, such as mutational profiles. In summary, the immune profiling described here could aid in refining MDS stratification, particularly in distinguishing patients who may benefit from immune‐modulating therapies such as IRAK4 inhibitors or NF‐κB‐targeting agents. Specifically, the enrichment of diverse immune‐related pathways in the HIC cluster suggests that these patients could be prime candidates for future immunotherapy strategies. Recent studies show that immune‐based stratification can accurately predict survival in MDS patients, with transcriptomic techniques paving the way for the integration of this tool in oncology [18, 52, 64, 65]. However, most immune‐modulating therapies for MDS are still in the preclinical phase [66, 67]. Future research should validate our insights and explore stratification for immune‐based target therapies in MDS.

## Conflicts of Interest

The authors declare that they have no known competing financial interests or personal relationships that could have influenced the work reported in this paper.

### Peer Review

The peer review history for this article is available at https://www.webofscience.com/api/gateway/wos/peer-review/10.1002/hon.70104.

## Supporting information

Supporting Information S1

## Data Availability

The data utilized in this study are accessible upon reasonable request to the corresponding author.

## References

[1] M. Cazzola , “Myelodysplastic Syndromes,” New England Journal of Medicine 383, no. 14 (October 2020): 1358–1374, 10.1056/nejmra1904794. 32997910

[2] J. J. Trowbridge and D. T. Starczynowski , “Innate Immune Pathways and Inflammation in Hematopoietic Aging, Clonal Hematopoiesis, and MDS,” Journal of Experimental Medicine [Internet], 218, no. 7 (July 2021), 10.1084/jem.20201544. PMC8210621 34129017

[3] C. T. R. Vegivinti , P. R. Keesari , S. Veeraballi , et al., “Role of Innate Immunological/Inflammatory Pathways in Myelodysplastic Syndromes and AML: A Narrative Review,” Experimental Hematology & Oncology 12, no. 1 (July 2023): 60, 10.1186/s40164-023-00422-1. 37422676 PMC10329313

[4] L. Nilsson , I. Astrand‐Grundström , K. Anderson , et al., “Involvement and Functional Impairment of the CD34(+)CD38(‐)Thy‐1(+) Hematopoietic Stem Cell Pool in Myelodysplastic Syndromes With Trisomy 8,” Blood 100, no. 1 (July 2002): 259–267, 10.1182/blood-2001-12-0188. 12070035

[5] T. Muto , C. S. Walker , K. Choi , et al., “Adaptive Response to Inflammation Contributes to Sustained Myelopoiesis and Confers a Competitive Advantage in Myelodysplastic Syndrome HSCs,” Nature Immunology 21, no. 5 (May 2020): 535–545, 10.1038/s41590-020-0663-z. 32313245 PMC7402480

[6] Williams N. , Lee J. , Moore L , et al. “Phylogenetic Reconstruction of Myeloproliferative Neoplasm Reveals Very Early Origins and Lifelong Evolution,” [Internet] preprint, bioRxiv (2020), 10.1101/2020.11.09.374710

[7] D. Van Egeren , J. Escabi , M. Nguyen , et al., “Reconstructing the Lineage Histories and Differentiation Trajectories of Individual Cancer Cells in Myeloproliferative Neoplasms,” Cell Stem Cell 28, no. 3 (March 2021): 514–523.e9, 10.1016/j.stem.2021.02.001. 33621486 PMC7939520

[8] L. Barreyro , T. M. Chlon , and D. T. Starczynowski , “Chronic Immune Response Dysregulation in MDS Pathogenesis,” Blood 132, no. 15 (October 2018): 1553–1560, 10.1182/blood-2018-03-784116. 30104218 PMC6182269

[9] M. T. Baldridge , K. Y. King , N. C. Boles , D. C. Weksberg , and M. A. Goodell , “Quiescent Haematopoietic Stem Cells Are Activated by IFN‐Gamma in Response to Chronic Infection,” Nature [Internet] 465, no. 7299 (June 2010): 793–797, 10.1038/nature09135. 20535209 PMC2935898

[10] V. Vallelonga , F. Gandolfi , F. Ficara , M. G. Della Porta , and S. Ghisletti , “Emerging Insights into Molecular Mechanisms of Inflammation in Myelodysplastic Syndromes,” Biomedicines 11, no. 10 (September 2023): 2613, 10.3390/biomedicines11102613. 37892987 PMC10603842

[11] L. C. Paracatu , D. A. Monlish , Z. J. Greenberg , et al., “Toll‐Like Receptor and Cytokine Expression Throughout the Bone Marrow Differs Between Patients With Low‐ and High‐Risk Myelodysplastic Syndromes,” Experimental Hematology 110 (June 2022): 47–59, 10.1016/j.exphem.2022.03.011. 35367529 PMC9590644

[12] A. G. de Matos , H. L. Ribeiro Junior , D. de Paula Borges , et al., “Interleukin‐8 and Nuclear Factor Kappa B Are Increased and Positively Correlated in Myelodysplastic Syndrome,” Medical Oncology 34, no. 10 (August 2017): 168, 10.1007/s12032-017-1023-1. 28856536

[13] V. R. Gummadi , A. Boruah , B. R. Ainan , et al., “Discovery of CA‐4948, an Orally Bioavailable IRAK4 Inhibitor for Treatment of Hematologic Malignancies,” ACS Medicinal Chemistry Letters 11, no. 12 (December 2020): 2374–2381, 10.1021/acsmedchemlett.0c00255. 33335659 PMC7734642

[14] S. Dimicoli , Y. Wei , C. Bueso‐Ramos , et al., “Overexpression of the Toll‐Like Receptor (TLR) Signaling Adaptor MYD88, But Lack of Genetic Mutation, in Myelodysplastic Syndromes,” PLoS One 8, no. 8 (August 2013): e71120, 10.1371/journal.pone.0071120. 23976989 PMC3744562

[15] X. Peng , X. Zhu , T. Di , et al., “The Yin‐Yang of Immunity: Immune Dysregulation in Myelodysplastic Syndrome With Different Risk Stratification,” Frontiers in Immunology 13 (September 2022): 994053, 10.3389/fimmu.2022.994053. 36211357 PMC9537682

[16] M. Kanehisa , M. Furumichi , Y. Sato , M. Kawashima , and M. Ishiguro‐Watanabe , “KEGG for Taxonomy‐Based Analysis of Pathways and Genomes,” Nucleic Acids Research 51, no. D1 (January 2023): D587–D592, 10.1093/nar/gkac963. 36300620 PMC9825424

[17] M. Kanehisa and S. Goto , “KEGG: Kyoto Encyclopedia of Genes and Genomes,” Nucleic Acids Research 28, no. 1 (January 2000): 27–30. 10592173 10.1093/nar/28.1.27 PMC102409

[18] A. M. Newman , C. L. Liu , M. R. Green , et al., “Robust Enumeration of Cell Subsets From Tissue Expression Profiles,” Nature Methods 12, no. 5 (May 2015): 453–457, 10.1038/nmeth.3337. 25822800 PMC4739640

[19] H. C. Wang and S. Fedoroff , “Karyology of Cells in Culture,” in Tissue Culture (Elsevier, 1973), 782–787.

[20] J. D. Khoury , E. Solary , O. Abla , et al., “The 5th Edition of the World Health Organization Classification of Haematolymphoid Tumours: Myeloid and Histiocytic/Dendritic Neoplasms,” Leukemia 36, no. 7 (July 2022): 1703–1719, 10.1038/s41375-022-01613-1. 35732831 PMC9252913

[21] M. Schneider , C. Rolfs , M. Trumpp , et al., “Activation of Distinct Inflammatory Pathways in Subgroups of LR‐MDS,” Leukemia 37, no. 8 (August 2023): 1709–1718, 10.1038/s41375-023-01949-2. 37420006 PMC10400420

[22] L. Chee , D. Ritchie , M. Ludford‐Menting , et al., “Dysregulation of Immune Cell and Cytokine Signalling Correlates With Clinical Outcomes in Myelodysplastic Syndrome (MDS),” European Journal of Haematology 108, no. 4 (April 2022): 342–353. 34963023 10.1111/ejh.13742

[23] A. Glenthøj , A. D. Ørskov , J. W. Hansen , S. R. Hadrup , C. O’Connell , and K. Grønbæk , “Immune Mechanisms in Myelodysplastic Syndrome,” International Journal of Molecular Sciences 17, no. 6 (June 2016): 944, 10.3390/ijms17060944. 27314337 PMC4926477

[24] I. Gañán‐Gómez , Y. Wei , D. T. Starczynowski , et al., “Deregulation of Innate Immune and Inflammatory Signaling in Myelodysplastic Syndromes,” Leukemia 29, no. 7 (July 2015): 1458–1469, 10.1038/leu.2015.69. 25761935 PMC4857136

[25] Y. Wei , S. Dimicoli , C. Bueso‐Ramos , et al., “Toll‐Like Receptor Alterations in Myelodysplastic Syndrome,” Leukemia 27, no. 9 (September 2013): 1832–1840, 10.1038/leu.2013.180. 23765228 PMC4011663

[26] L. Suárez , M. B. Vidriales , J. García‐Laraña , et al., “CD34+ Cells From Acute Myeloid Leukemia, Myelodysplastic Syndromes, and Normal Bone Marrow Display Different Apoptosis and Drug Resistance‐Associated Phenotypes,” Clinical Cancer Research 10, no. 22 (November 2004): 7599–7606, 10.1158/1078-0432.ccr-04-0598. 15569991

[27] T. Xing , W. L. Yao , H. Y. Zhao , et al., “Bone Marrow Macrophages Are Involved in the Ineffective Hematopoiesis of Myelodysplastic Syndromes,” Journal of Cellular Physiology 239, no. 2 (February 2024): e31129, 10.1002/jcp.31129. 38192063

[28] S. J. Xu , Z. H. Shao , R. Fu , et al., “Subtype and Functional Biomarker Changes of NK Cells in Peripheral Blood of Patients With Myelodysplastic Syndrome,” Zhongguo Shi Yan Xue Ye Xue Za Zhi 25, no. 3 (June 2017): 832–836. 28641645 10.7534/j.issn.1009-2137.2017.03.036

[29] M. Carlsten and M. Järås , “Natural Killer Cells in Myeloid Malignancies: Immune Surveillance, NK Cell Dysfunction, and Pharmacological Opportunities to Bolster the Endogenous NK Cells,” Frontiers in Immunology 10 (October 2019): 2357, 10.3389/fimmu.2019.02357. 31681270 PMC6797594

[30] T. Braun , G. Carvalho , A. Coquelle , et al., “NF‐kappaB Constitutes a Potential Therapeutic Target in High‐Risk Myelodysplastic Syndrome,” Blood 107, no. 3 (February 2006): 1156–1165, 10.1182/blood-2005-05-1989. 16223780

[31] J. Fang , L. C. Bolanos , K. Choi , et al., “Ubiquitination of hnRNPA1 by TRAF6 Links Chronic Innate Immune Signaling With Myelodysplasia,” Nature Immunology 18, no. 2 (February 2017): 236–245, 10.1038/ni.3654. 28024152 PMC5423405

[32] G. W. Rhyasen , L. Bolanos , J. Fang , et al., “Targeting IRAK1 as a Therapeutic Approach for Myelodysplastic Syndrome,” Cancer Cell 24, no. 1 (July 2013): 90–104, 10.1016/j.ccr.2013.05.006. 23845443 PMC3711103

[33] M. Daher , J. E. Hidalgo Lopez , J. K. Randhawa , et al., “An Exploratory Clinical Trial of Bortezomib in Patients With Lower Risk Myelodysplastic Syndromes,” American Journal of Hematology 92, no. 7 (July 2017): 674–682, 10.1002/ajh.24746. 28370157 PMC5580683

[34] H. Chen , M. Li , R. A. Campbell , et al., “Interference With Nuclear Factor Kappa B and C‐Jun NH2‐terminal Kinase Signaling by TRAF6C Small Interfering RNA Inhibits Myeloma Cell Proliferation and Enhances Apoptosis,” Oncogene 25, no. 49 (October 2006): 6520–6527, 10.1038/sj.onc.1209653. 16702955

[35] G. Garcia‐Manero , E. S. Winer , D. J. DeAngelo , et al., “S129: Takeaim Leukemia‐ A Phase 1/2a Study of the Irak4 Inhibitor Emavusertib (Ca‐4948) as Monotherapy or in Combination With Azacitidine or Venetoclax in Relapsed/refractory Aml or Mds,” HemaSphere 6 (June 2022): 30–31, 10.1097/01.hs9.0000843408.31385.3f.

[36] M. E. Varney , K. Melgar , M. Niederkorn , M. Smith , L. Barreyro , and D. T. Starczynowski , “Deconstructing Innate Immune Signaling in Myelodysplastic Syndromes,” Experimental Hematology 43, no. 8 (August 2015): 587–598, 10.1016/j.exphem.2015.05.016. 26143580 PMC4635673

[37] L. C. Paracatu and L. G. Schuettpelz , “Contribution of Aberrant Toll Like Receptor Signaling to the Pathogenesis of Myelodysplastic Syndromes,” Frontiers in Immunology 11 (June 2020): 1236, 10.3389/fimmu.2020.01236. 32625214 PMC7313547

[38] L. Chen , L. Zheng , P. Chen , and G. Liang , “Myeloid Differentiation Primary Response Protein 88 (MyD88): The Central Hub of TLR/IL‐1R Signaling,” Journal of Medicinal Chemistry 63, no. 22 (November 2020): 13316–13329, 10.1021/acs.jmedchem.0c00884. 32931267

[39] C. Li , J. Zienkiewicz , and J. Hawiger , “Interactive Sites in the MyD88 Toll/Interleukin (IL) 1 Receptor Domain Responsible for Coupling to the IL1beta Signaling Pathway,” Journal of Biological Chemistry 280, no. 28 (July 2005): 26152–26159, 10.1074/jbc.m503262200. 15849357

[40] J. Vaquero , J. S. Campbell , J. Haque , et al., “Toll‐Like Receptor 4 and Myeloid Differentiation Factor 88 Provide Mechanistic Insights Into the Cause and Effects of Interleukin‐6 Activation in Mouse Liver Regeneration,” Hepatology 54, no. 2 (August 2011): 597–608, 10.1002/hep.24420. 21574169 PMC4247827

[41] M. Jongen‐Lavrencic , H. R. Peeters , H. Rozemuller , et al., “IL‐6‐Induced Anaemia in Rats: Possible Pathogenetic Implications for Anemia Observed in Chronic Inflammations,” Clinical and Experimental Immunology 103, no. 2 (February 1996): 328–334, 10.1046/j.1365-2249.1996.d01-622.x. 8565320 PMC2200351

[42] J. Krajewski , C. Batmunkh , W. Jelkmann , and T. Hellwig‐Bürgel , “Interleukin‐1β Inhibits the Hypoxic Inducibility of the Erythropoietin Enhancer by Suppressing Hepatocyte Nuclear Factor‐4α,” Cellular and Molecular Life Sciences 64, no. 7–8 (April 2007): 989–998, 10.1007/s00018-007-6561-9. 17372675 PMC11136359

[43] R. T. Jr Means , E. N. Dessypris , and S. B. Krantz , “Inhibition of Human Erythroid Colony‐Forming Units by Interleukin‐1 is Mediated by Gamma Interferon,” Journal of Cellular Physiology 150, no. 1 (January 1992): 59–64, 10.1002/jcp.1041500109. 1730787

[44] N. Ramachandra , M. Gupta , L. Schwartz , et al., “Role of IL8 in Myeloid Malignancies,” Leukemia and Lymphoma 64, no. 11 (November 2023): 1742–1751, 10.1080/10428194.2023.2232492. 37467070

[45] K. A. Lord , B. Hoffman‐Liebermann , and D. A. Liebermann , “Nucleotide Sequence and Expression of a cDNA Encoding MyD88, a Novel Myeloid Differentiation Primary Response Gene Induced by IL6,” Oncogene 5, no. 7 (July 1990): 1095–1097. 2374694

[46] Y. Zhang , M. Jones , A. McCabe , G. M. Winslow , D. Avram , and K. C. MacNamara , “MyD88 Signaling in CD4 T Cells Promotes IFN‐γ Production and Hematopoietic Progenitor Cell Expansion in Response to Intracellular Bacterial Infection,” Journal of Immunology 190, no. 9 (May 2013): 4725–4735, 10.4049/jimmunol.1203024. PMC3633622 23526822

[47] K. Fiedler , E. Kokai , S. Bresch , and C. Brunner , “MyD88 is Involved in Myeloid as Well as Lymphoid Hematopoiesis Independent of the Presence of a Pathogen,” American Journal of Blood Research 3, no. 2 (May 2013): 124–140. 23675564 PMC3649812

[48] M. Samba‐Mondonga , A. Calvé , F. A. Mallette , and M. M. Santos , “MyD88 Regulates the Expression of SMAD4 and the Iron Regulatory Hormone Hepcidin,” Frontiers in Cell and Developmental Biology 6 (August 2018): 105, 10.3389/fcell.2018.00105. 30234111 PMC6127602

[49] A. Layoun , M. Samba‐Mondonga , G. Fragoso , A. Calvé , and M. M. Santos , “MyD88 Adaptor Protein is Required for Appropriate Hepcidin Induction in Response to Dietary Iron Overload in Mice,” Frontiers in Physiology 9 (March 2018): 159, 10.3389/fphys.2018.00159. 29556203 PMC5845127

[50] O. Adachi , T. Kawai , K. Takeda , et al., “Targeted Disruption of the MyD88 Gene Results in Loss of IL‐1‐ and IL‐18‐Mediated Function,” Immunity 9, no. 1 (July 1998): 143–150, 10.1016/s1074-7613(00)80596-8. 9697844

[51] M. E. Varney , M. Niederkorn , H. Konno , et al., “Loss of Tifab, a del(5q) MDS Gene, Alters Hematopoiesis Through Derepression Of Toll‐Like Receptor‐TRAF6 Signaling,” Journal of Experimental Medicine 212, no. 11 (October 2015): 1967–1985, 10.1084/jem.20141898. 26458771 PMC4612089

[52] Y. H. Wang , H. A. Hou , C. C. Lin , et al., “A CIBERSORTx‐Based Immune Cell Scoring System Could Independently Predict the Prognosis of Patients With Myelodysplastic Syndromes,” Blood Advances 5, no. 22 (November 2021): 4535–4548, 10.1182/bloodadvances.2021005141. 34614508 PMC8759137

[53] J. Bennett , C. Ishikawa , P. Agarwal , et al., “Paralog‐specific Signaling by IRAK1/4 Maintains MyD88‐Independent Functions in MDS/AML,” Blood 142, no. 11 (September 2023): 989–1007, 10.1182/blood.2022018718. 37172199 PMC10517216

[54] R. D. Parrondo , M. Iqbal , R. Von Roemeling , C. Von Roemeling , and H. W. Tun , “IRAK‐4 Inhibition: Emavusertib for the Treatment of Lymphoid and Myeloid Malignancies,” Frontiers in Immunology 14 (October 2023): 1239082, 10.3389/fimmu.2023.1239082. 37954584 PMC10637517

[55] A. Wojtuszkiewicz , G. J. Schuurhuis , F. L. Kessler , et al., “Exosomes Secreted by Apoptosis‐Resistant Acute Myeloid Leukemia (AML) Blasts Harbor Regulatory Network Proteins Potentially Involved in Antagonism of Apoptosis,” Molecular & Cellular Proteomics 15, no. 4 (April 2016): 1281–1298, 10.1074/mcp.m115.052944. 26801919 PMC4824855

[56] A. M. Abdul‐Aziz , M. S. Shafat , T. K. Mehta , et al., “MIF‐Induced Stromal PKCβ/IL8 is Essential in Human Acute Myeloid Leukemia,” Cancer Research 77, no. 2 (January 2017): 303–311, 10.1158/0008-5472.can-16-1095. 27872094

[57] J. Schanz , C. Steidl , C. Fonatsch , et al., “Coalesced Multicentric Analysis of 2,351 Patients With Myelodysplastic Syndromes Indicates an Underestimation of Poor‐Risk Cytogenetics of Myelodysplastic Syndromes in the International Prognostic Scoring System,” Journal of Clinical Oncology 29, no. 15 (May 2011): 1963–1970, 10.1200/jco.2010.28.3978. 21519021 PMC4874202

[58] J. Neukirchen , M. Lauseker , B. Hildebrandt , et al., “Cytogenetic Clonal Evolution in Myelodysplastic Syndromes is Associated With Inferior Prognosis,” Cancer 123, no. 23 (December 2017): 4608–4616, 10.1002/cncr.30917. 28746789

[59] I. Sayers , D. Thakker , C. Billington , et al., “Interleukin‐1 Receptor‐Associated Kinase 4 (IRAK4) is a Critical Regulator of Inflammatory Signalling Through Toll‐Like Receptors 4 and 7/8 in Murine and Human Lungs,” British Journal of Pharmacology 181, no. 22 (November 2024): 4647–4657, 10.1111/bph.16509. 39137914 PMC7618454

[60] X. Li , “IRAK4 in TLR/IL‐1R Signaling: Possible Clinical Applications,” European Journal of Immunology 38, no. 3 (March 2008): 614–618, 10.1002/eji.200838161. 18286571

[61] S. De , F. Karim , E. Kiessu , et al., “Mechanism of Dysfunction of Human Variants of the IRAK4 Kinase and a Role for Its Kinase Activity in Interleukin‐1 Receptor Signaling,” Journal of Biological Chemistry 293, no. 39 (September 2018): 15208–15220, 10.1074/jbc.ra118.003831. 30115681 PMC6166721

[62] M. Koziczak‐Holbro , C. Joyce , A. Glück , et al., “IRAK‐4 Kinase Activity is Required for Interleukin‐1 (IL‐1) Receptor‐ and Toll‐Like Receptor 7‐Mediated Signaling and Gene Expression,” Journal of Biological Chemistry 282, no. 18 (May 2007): 13552–13560. 17337443 10.1074/jbc.M700548200

[63] H. L. Ribeiro Junior , P. G. Gonçalves , D. A. Moreno , et al., “Discrepancy in Transcriptomic Profiling Between CD34 + Stem Cells and Primary Bone Marrow Cells in Myelodysplastic Neoplasm,” Leukemia Research 129, no. 107071 (June 2023): 107071. 37004280 10.1016/j.leukres.2023.107071

[64] Y. H. Wang , C. C. Lin , C. Y. Yao , et al., “Immune Signatures of Bone Marrow Cells Can Independently Predict Prognosis in Patients With Myelodysplastic Syndrome,” British Journal of Haematology 196, no. 1 (January 2022): 156–168, 10.1111/bjh.17837. 34536013

[65] D. Aran , Z. Hu , and A. J. Butte , “xCell: Digitally Portraying the Tissue Cellular Heterogeneity Landscape,” Genome Biology 18, no. 1 (November 2017): 220, 10.1186/s13059-017-1349-1. 29141660 PMC5688663

[66] R. J. Stubbins , H. Cherniawsky , and A. Karsan , “Cellular and Immunotherapies for Myelodysplastic Syndromes,” Seminars in Hematology 61, no. 6 (December 2024): 397–408, 10.1053/j.seminhematol.2024.09.006. 39426936

[67] A. M. Brunner , “Novel Immune Directed Therapies in Myelodysplastic Syndromes and Acute Myeloid Leukemia,” Current Opinion in Hematology 30, no. 2 (March 2023): 38–44, 10.1097/moh.0000000000000749. 36728945 PMC10017015

