# Supplementary material for: Clustering Based on Innate Immunity Reveals Differential Dysregulation Based on Disease Severity in Myelodysplastic Neoplasms
Source: Hematol Oncol. 2025 May 21;43(3):e70104. doi: 10.1002/hon.70104 (PMC12094220; doi:10.1002/hon.70104)
Supplement: Supplementary file 1 — Supporting Information S1 [file HON-43-e70104-s001.docx]

**ONLINE SUPPLEMENT**

**Clustering Based on Innate Immunity Reveals Differential Dysregulation Based on Disease Severity in Myelodysplastic Neoplasms**

Pedro Robson Costa Passos^a,b^; Andréa Alcântara Vieira^a,b,d^*; Renata Pinheiro Martins de Melo^a,b^; Ronald Feitosa Pinheiro Filho^ab^; Leonardo Guimarães Sampaio^a,b^; Hermano Vinnicius Gomes dos Santos^a,b^; Letícia Rodrigues Sampaio^a,b,d^; João Victor Caetano Goes^a,b,e^; Sílvia Maria Meira Magalhães^a,b,c,d,e^; Ronald Feitosa Pinheiro^a,b,c,d,e^

*Equal contributions to this paper

a. Laboratory of Cancer Cytogenetics, Federal University of Ceará, Fortaleza, Ceará, Brazil.

b. Research Center for Drug Development (NPDM), Fortaleza, Ceará, Brazil.

c. Department of Clinical Medicine, Federal University of Ceará, Fortaleza, Ceará, Brazil.

d. Postgraduate Program in Medical Sciences, Federal University of Ceará, Fortaleza, Ceará, Brazil.

e. Postgraduate Program in Pathology, Federal University of Ceará, Fortaleza, Ceará, Brazil.

**Summary**

[Table S1 - Individual patient characteristics in the in-house cohort 3](#_jh3yqwrhlk8z)

[Table S2. Specific parameters of differentially expressed genes. 8](#_xev07035pp4r)

[Table S3 - All initial analyses conducted on clinical, morphological and molecular data of the in-house cohort 11](#_rodynvwozuh7)

#

# Table S1 - Individual patient characteristics in the in-house cohort

| **Patient** | **Sex** | **Age** | **Cellular** | **Karyotype** | **WHO_2022** | **IPSS_R** | **Transfusion_Dependency** |
| --- | --- | --- | --- | --- | --- | --- | --- |
| 1 | F | 81 | - | 46,XX[5] | MDS-LB-RS | Low | Yes |
| 2 | F | 46 | Hypercellular | 46,XX[8] | MDS-LB | Low | Yes |
| 3 | M | 86 | Hypercellular | 46,XY[25] | MDS-LB | Intermediate | Yes |
| 4 | M | 63 | - | Complex | MDS-IB | High | Yes |
| 5 | M | 66 | Hypercellular | - | MDS-LB | - | Yes |
| 6 | M | 32 | Hypocellular | del(5), del(17) | MDS-LB | Low | No |
| 7 | M | 92 | Hypercellular | No metaphases | MDS-LB | - | No |
| 8 | M | 85 | Hypercellular | 46,XY[20] | MDS-LB | Low | No |
| 9 | M | 77 | - | Complex | MDS-LB | Very High | Yes |
| 10 | F | 44 | - | del(5), del(11) | MDS-LB | Low | Yes |
| 11 | M | 47 | Normocellular | +mar, del(5) | MDS-LB | Low | Yes |
| 12 | M | 81 | Hypercellular | 46,XY[20] | MDS-LB | Intermediate | Yes |
| 13 | M | 79 | Normocellular | 46,XY[6] | MDS-LB | Low | No |
| 14 | M | 71 | Hypercellular | 46,XY[17] | MDS-LB-RS | Low | No |
| 15 | M | 68 | Hypercellular | No metaphases | MDS-IB | - | Yes |
| 16 | F | 72 | - | - | MDS-IB | - | Yes |
| 17 | F | 59 | - | No metaphases | MDS-LB | - | Yes |
| 18 | F | 84 | - | No metaphases | MDS-LB-RS | - | No |
| 19 | M | 78 | - | - | MDS-LB | - | - |
| 20 | F | 73 | Hypercellular | 46,XX[20] | MDS-LB-RS | Low | Yes |
| 21 | F | 70 | Hypercellular | - | MDS-LB-RS | - | Yes |
| 22 | M | 79 | - | del(5), del(11) | MDS-LB | Intermediate | Yes |
| 23 | M | 25 | Hypocellular | 46,XY[20] | MDS-h | Low | Yes |
| 24 | F | 60 | Normocellular | 45,X,-X | MDS-IB | Very High | Yes |
| 25 | M | 60 | - | +15 | MDS-LB-RS | Intermediate | Yes |
| 26 | M | 40 | Hypocellular | Complex | MDS-h | Intermediate | No |
| 27 | F | 87 | Hypercellular | t(4;11), +mar | MDS-LB | Intermediate | No |
| 28 | F | 77 | Hypercellular | 46,XX[15] | MDS-LB | Very Low | No |
| 29 | F | 74 | Hypercellular | No metaphases | MDS-LB | - | Yes |
| 30 | M | 59 | - | No metaphases | MDS-LB | - | No |
| 31 | M | 61 | Hypercellular | No metaphases | MDS-LB-RS | - | No |
| 32 | F | 53 | Hypercellular | 46,XX[20] | MDS-LB | Low | No |
| 33 | F | 63 | Hypocellular | 46,XY[10] | MDS-LB | Low | No |
| 34 | M | 76 | Hypercellular | No metaphases | MDS-LB-RS | - | No |
| 35 | M | 81 | Hypocellular | 46,XY[18] | MDS-LB | Low | No |
| 36 | M | 92 | - | 46,XY,del(5)(q32)[13]/46,XY[17] | MDS-Del(5q) | Low | - |
| 37 | M | 71 | Normocellular | 46,XY[20] | MDS-IB | Intermediate | Yes |
| 38 | F | 75 | - | 46,XX[20] | MDS-LB-RS | Low | - |
| 39 | M | 75 | Hypercellular | 92,XXYY<4n>[4]/46,XY[8] | MDS-IB | High | - |
| 40 | M | 83 | Hypercellular | 92,XXYY[5]/46,XY[15] | MDS-LB | Very Low | No |
| 41 | M | 71 | Hypercellular | 46,XY[20] | MDS-LB | Low | Yes |
| 42 | F | 25 | Hypocellular | 46,XX[20] | MDS-h | Intermediate | - |
| 43 | F | 78 | - | 46,XX[20] | MDS-LB | Low | No |
| 44 | M | 79 | - | 46,XY[20] | MDS-IB | Intermediate | - |
| 45 | F | 81 | - | 47,XX,+8[9]/47,XX,+8,del(20)(q12)[5]/46,XX[6] | MDS-LB-RS | Intermediate | - |
| 46 | F | 83 | - | 46,XX[12] | MDS-IB | High | - |
| 47 | F | 81 | Hypercellular | 46,XX[20] | MDS-LB | Very Low | No |
| 48 | M | 89 | - | 46,XY,t(5;19)(q13.2;q13.4)[3]/46,XY,t(5;19)(q13.2;q13.4),t(8;21)(q21.3;q22.12)[3]/46,XY,del(X)(q21),t(5;19)(q13.2;q13.4),t(8;21)(q21.3;q22.12)[5]/46,XY[9] | MDS-IB | Very high | - |
| 49 | F | 46 | Hypercellular | 46,XX[20] | MDS-LB | Low | No |
| 50 | F | 93 | - | 46,XX,+8[12]/46,XX[8] | MDS-IB | Very high | - |
| 51 | M | 84 | - | 46,XY[20] | MDS-IB | High | - |
| 52 | F | 81 | Hypercellular | 46,XX[20] | MDS-LB-RS | Low | - |
| 53 | M | 87 | - | 46,XY[20] | MDS-LB | Low | No |
| 54 | F | 49 | - | 46,XX[4] | MDS-LB | Low | - |
| 55 | M | 76 | Hypercellular | 46,XY,del(5)(q31)[2]/46,XY,del(5)(q31),-7,+8[16] | MDS-IB | Very high | - |
| 56 | F | 85 | - | 46,XX[20] | MDS-IB | Intermediate | - |
| 57 | F | 48 | Hypocellular | 46,XX[20] | MDS-h | Intermediate | - |
| 58 | M | 73 | Hypercellular | 46,XY[20] | MDS-IB | High | - |
| 59 | F | 91 | - | 46,XX[20] | MDS-IB | High | Yes |
| 60 | M | 65 | Hypercellular | 46,XY[20] | MDS-f | High | - |
| 61 | F | 87 | - | 46,XX,del(5)(q14)[5]/46,XX[15] | MDS-IB | Very high | - |
| 62 | F | 86 | - | Trisomy 8 | MDS-IB | High | - |
| 63 | M | 86 | Hypercellular | 46,XY[20] | MDS-LB-RS | Low | - |
| 64 | F | 44 | Hypercellular | 46,XX[20] | MDS-LB | Low | - |
| 65 | M | 86 | - | 92,XY[3]/46,XY[4] | MDS-LB-RS | High | - |
| 66 | M | 69 | Hypercellular | 43,XY,del(3)(p22),del(4)(q21),-5,-6,-7,+add(16)(p13),add(20)(p13),+mar[20] | MDS-biTP53 | Very high | - |
| 67 | M | 81 | Hypercellular | 46,XY[18] | MDS-LB | Low | - |
| 68 | F | 55 | - | 92-184,XX[8]/46,XX[20] | MDS-IB | High | No |
| 69 | M | 69 | - | 46,XY[15] | MDS-IB | Very high | - |
| 70 | F | 77 | Hypercellular | 46,XX[20] | MDS-LB | Low | Yes |
| 71 | F | 65 | Normocellular | 46,XX, 16qh+[20] | MDS-LB | Low | No |
| 72 | F | 64 | Hypercellular | 46,XX[20] | MDS-IB | High | Yes |
| 73 | F | 71 | Hypercellular | 46,XX[10] | MDS-IB | High | No |
| 74 | F | 79 | - | 46,XX,del(5)(q13)[8]/46,XX[12] | MDS-IB | Very High | - |
| 75 | F | 86 | - | 46,XX,del(5)(q14)[20] | MDS-Del(5q) | Low | - |
| 76 | F | 65 | Hypercellular | 47,XX,+mar[12] | MDS-IB | Very high | - |
| 77 | F | 69 | - | 45,XX,-5,del(5)(q31)[3]/45,XX,-5,del(5)(q31),der(1)t(1;8)(p36.3;q24)[16]/46,XX[1] | AML-RM | - | - |
| 78 | M | 79 | - | 46,XY[20] | AML-RM | - | No |
| 79 | M | 76 | - | 46,XY[20] | AML-RM | - | No |
| 80 | F | 82 | - | 46,XX,del(3)(q21),-5,+6,+8,i(11)(q10),add(16)(p13.3),add(17)(p13),+mar[20] | AML-RM | - | No |
| 81 | M | 75 | - | 46,XY[20] | AML-RM | - | - |
| 82 | M | 81 | - | 45,X,-Y[20] | AML-RM | - | - |

*MDS-LB* myelodysplastic neoplasm with low blasts, *MDS-LB-RS* myelodysplastic neoplasm with low blasts and ring sideroblasts, *MDS-IB* myelodysplastic neoplasm with increased blasts, *MDS-biTP5*3 myelodysplastic neoplasm with biallelic TP53 inactivation, *MDS-del(5q)* with low blasts and isolated 5q deletion, *MDS-f* myelodysplastic neoplasm with fibrosis, *MDS-h* myelodysplastic neoplasm hypoplastic, *AML-RM* acute myeloid leukemia related to myelodysplasia

#

# Table S2. Specific parameters of differentially expressed genes.

| Differentially expressed gene | log_2_FC | Adjusted p-value |
| --- | --- | --- |
| LOC100129518 /// SOD2 | -1.5411558 | 1.280434e-10 |
| PHLDA1 | -1.3021154 | 7.443121e-10 |
| TNFAIP3 | -1.2788395 | 2.413098e-09 |
| CXCL3 | -1.4627660 | 3.139574e-09 |
| IER3 | -1.8186600 | 6.958330e-09 |
| METRNL | -1.2702831 | 7.050413e-09 |
| CD83 | -1.0481658 | 1.005862e-08 |
| C15orf48 | -1.1825483 | 2.143119e-08 |
| LOC145474 | -1.4495507 | 2.400755e-08 |
| CCL3 /// CCL3L1 /// CCL3L3 | -1.7840330 | 3.750979e-08 |
| CCL4 | -1.8270561 | 4.587273e-08 |
| SGK1 | -1.3804517 | 1.113414e-07 |
| IL1RN | -1.5161077 | 1.140032e-07 |
| GADD45B | -1.1482539 | 1.435760e-07 |
| PLAUR | -1.0610542 | 2.299372e-07 |
| PTGS2 | -1.2905595 | 4.140268e-07 |
| BIRC3 | -1.0951739 | 5.689883e-07 |
| HCAR3 | -1.2272069 | 8.432251e-07 |
| PTX3 | -1.3596312 | 1.529926e-06 |
| IL10RA | -1.0718252 | 2.459884e-06 |
| CXCL8 | -1.4626827 | 3.729357e-06 |
| ADM | -1.1201587 | 5.405693e-06 |
| CCL20 | -1.0265174 | 7.102215e-06 |
| DUSP2 | -1.0608660 | 7.281804e-06 |
| AQP9 | -1.0566044 | 9.427739e-06 |
| DDIT4 | -1.1921030 | 1.138197e-05 |
| GEM | -1.0556805 | 3.022857e-05 |
| BRE-AS1 | -1.1280510 | 3.592502e-05 |
| GPR183 | -1.0950059 | 9.152654e-05 |
| EGR3 | -1.1022972 | 2.981720e-04 |
| RPL31 /// TBC1D8 | 1.2890986 | 3.979368e-04 |
| CXCL10 | -1.0126345 | 5.087990e-04 |
| SERPINB2 | -1.0330391 | 5.407464e-04 |

*log-FC* log-fold change

# Table S3 - All group comparisons conducted on clinical, morphological and molecular data of all patients on the in-house cohort based on gene expression data

| Variable | Levels | *IRAK1* (p-value) | *IRAK2* (p-value) | *IRAK4* (p-value) | *MYD88* (p-value) | *TRAF6* (p-value) | *NFKB1* (p-value |
| --- | --- | --- | --- | --- | --- | --- | --- |
| Age | < 60 years, 60 - 80 years, >80 years | 0.801 | 0.401 | 0.243 | 0.318 | 0.917 | 0.315 |
| Hemoglobin count on peripheral blood | < 8 g/dL, 8–10 g/dL, and > 10 g/dL | 0.856 | 0.127 | 0.415 | 0.026* | 0.324 | 0.763 |
| Absolute neutrophil count on peripheral blood | < 800 mm³ and ≥ 800 mm³ | 0.844 | 0.176 | 0.127 | 0.667 | 0.101 | 0.778 |
| Platelets count on peripheral blood | < 50,000; 50,000 - < 100,000; and ≥ 100,000 | 0.625 | 0.182 | 0.249 | 0.363 | 0.855 | 0.435 |
| Percentage of blasts in bone marrow aspirate | ≤ 2%, > 2%–< 5%, 5–10%, and > 10% | 0.024* | 0.004* | 0.036* | 0.015* | 0.198 | 0.054 |
| Presence of ring sideroblasts in bone marrow biopsy | Yes, no | 0.591 | 0.620 | 0.589 | 0.672 | 0.757 | 0.439 |
| Dyserythropoiesis | Yes, no | 0.955 | 0.878 | 0.291 | 0.418 | 0.584 | 0.889 |
| Dysgranulopoiesis | Yes, no | 0.695 | 0.744 | 0.418 | 0.343 | 0.646 | 0.767 |
| Dysmegakaryopoiesis | Yes, no | 0.906 | 0.201 | 0.901 | 0.134 | 0.821 | 0.652 |
| Fibrosis on bone marrow biopsy | Yes, no | 0.612 | 0.490 | 0.170 | 0.765 | 0.506 | 0.969 |
| Cytogenetics | Normal, altered non-complex, complex | 0.367 | 0.721 | 0.312 | 0.071 | 0.367 | 0.950 |
| Disease subtype | MDS-del5q, MDS-LB-RS, MDS-LB, MDS-IB1- MDS-IB2, MDS-f, MDS-h, MDS-biTP53, AML-MR | 0.164 | 0.067 | 0.554 | 0.015* | 0.229 | 0.751 |

Comparisons involving more than three factors were analyzed using the Kruskal-Wallis test with Dunn’s post-hoc adjustment. Comparisons between two factors were performed using the Wilcoxon test. *MDS-LB* myelodysplastic neoplasm with low blasts, *MDS-LB-RS* myelodysplastic neoplasm with low blasts and ring sideroblasts, *MDS-IB* myelodysplastic neoplasm with increased blasts, *MDS-biTP5*3 myelodysplastic neoplasm with biallelic TP53 inactivation, *MDS-del(5q)* with low blasts and isolated 5q deletion, *MDS-f* myelodysplastic neoplasm with fibrosis, *MDS-h* myelodysplastic neoplasm hypoplastic, *AML-RM* acute myeloid leukemia related to myelodysplasia, *IPSS-R* revised international prognostic scoring system
